# Hydrogen Detection with SAW Polymer/Quantum Dots Sensitive Films

**DOI:** 10.3390/s19204481

**Published:** 2019-10-16

**Authors:** Izabela Constantinoiu, Cristian Viespe

**Affiliations:** National Institute for Laser, Plasma and Radiation Physics, Laser Department, Atomistilor #409, 077125 Bucharest-Magurele, Romania; izabela.constantinoiu@inflpr.ro

**Keywords:** gas sensor, composite, surface acoustic wave, quantum dots, polymer, hydrogen sensor, SAW sensor, gas detection

## Abstract

Regarding the use of hydrogen as a fuel, it is necessary to measure its concentration in air at room temperature. In this paper, sensitive composite films have been developed for surface acoustic wave (SAW) sensors, using quantum dots (QDs) and polymers. Si/SiO_2_ QDs were used due to having a high specific surface area, which considerably improves the sensitivity of the sensors compared to those that only have a polymer. Si/SiO_2_ QDs were obtained by laser ablation and analyzed by X-ray diffraction and transmission electron microscopy (TEM). Two types of polymers were used: polydimethylsiloxane (PDMS) and polymethylmethacrylate (PMMA). Polymer and polymer with QDs compositions were deposited on the sensor substrate by drop casting. A heat treatment was performed on the films at 80 °C with a thermal dwell of two hours. The sensors obtained were tested at different hydrogen concentrations at room temperature. A limit of detection (LOD) of 452 ppm was obtained by the sensor with PDMS and Si/SiO_2_ QDs, which was heat treated. The results demonstrated the potential of using QDs to improve the sensitivity of the SAW sensors and to achieve a heat treatment that increases its adsorption capacity of the gas molecules.

## 1. Introduction

Surface Acoustic Wave (SAW) sensors present a great opportunity in the field of gas detection due to their increased sensitivity, the possibility of wireless operation, small size, fast response, and the ability of integration with different receptor materials [1]. These characteristics allow the use of SAW sensors for both different gases (volatile organic compounds, toxic gases, explosives) as well as for biological analytes, such as for biological analytes, proteins, cancer cells, etc. [1]. They work on the principle of transduction, in which an electrical input signal turns into a mechanical wave, then again turns into an electrical signal [2].

The parameters which by their abnormal values can lead to the disturbance of the waves crossing the sensor and producing a frequency shift of the SAW sensors, can be temperature, pressure, mass loading, and acoustic-electric effect [2]. The mass loading effect is one of the most interesting response mechanisms for this type of sensors. This effect is based on the interaction at the level of the sensitive film with the entity that is required to be detected. This mass accumulation at the film level produces a frequency deviation that can be measured. Ideally, the signal obtained should be as high as possible at the lowest analyte concentration.

Until now, several types of materials have been used to develop sensitive films. These include polymers [3,4] metal oxides [5,6], and composite materials [7,8,9]. Each of these types of materials can improve the sensibility of the film, but by using composite materials we can obtain both new material properties and improved properties. Thus, in this paper, two types of sensitive films will be addressed and compared: polymer sensitive films and composite sensitive films made from polymer and quantum dots (QDs) and also the influence of a heat treatment on the sensitive properties of the films will be studied. 

It is known that the main advantage of using nanoparticles in this field is given by their large specific surface, which is inversely proportional to their size [7]. QDs are crystalline nanoparticles with small sizes (1–12 nm) [10]. Considering this aspect, the use of QDs for the synthesis of sensitive films represents a relatively new and interesting direction of research [11,12]. One of the synthesis methods for QDs, which ensures the control of the morphology and high purity, is pulsed laser deposition (PLD) [13,14]. 

Hydrogen is one of the gases for which there is still a great interest in the development of sensors capable of detecting it at the lowest concentrations [15]. It is one of the most abundant elements in the universe and the first element in the periodic table. It is a colorless, odorless, and insipid gas, in normal conditions. It is used in various domains like ammonia synthesis, fat hydrogenation, as well as rocket and car fuel [16]. If it is used as car fuel then it can reduce pollution, but it needs careful monitoring due to its flammable nature. 

As thin films for hydrogen detection, palladium is noted for its ability to dissociate the hydrogen molecule [9]. Also, oxidic films [5], as well as composite films [17,18,19] are often used in the development of hydrogen sensors. Polymers are noted as having a good sensitivity to room temperature, compared to the oxide materials, which most often require high temperature to obtain the signal. Instead, polymers age faster and offer a shorter lifetime in the sensors, compared to the oxide materials [20,21]. Thus, the composites of polymers and oxide materials can be a variant for optimizing the properties of the two types of materials, in the field of sensors [21].

Polymethylmethacrylate (PMMA) polymer has good properties for use in electronic and storage devices and sensors. Also, it is noted to have a good thermal stability under different environmental conditions [19,22]. Polydimethylsiloxane (PDMS) is a polymer used because of its low cost, high thermal expansion coefficient, and good adhesion to silicon. It is also used because of its dielectric properties, even in hydrogen sensors [9,23,24].

## 2. Materials and Methods

The SAW sensor was a ‘delay-line’ type, based on a quartz substrate (ST cut, X propagation, 10 mm wide, 38 mm long, 0.5 mm thick, and 3158 m/s velocity), cut in a parallelogram geometry to reduce the effect of SAW reflections from the edges of the piezoelectric substrate [25], with an oscillating frequency of about 69 MHz. The gold IDTs with ~150 nm thickness, were deposited using standard photolithographic techniques onto 10 nm thick chromium layers to ensure the adhesion of gold on the quartz. The IDTs have a ‘double-comb’ configuration, which consisted of 50 electrode pairs with a 2500 µm acoustic aperture and a 45.2 µm wavelength.

Si/SiO_2_ QDs were produced by laser ablation method, using an experimental setup made of a stainless-steel chamber, target system, gas handling, focusing lens, and collector. The experiments were conducted in a flow of argon and helium (1 l/min flow rate, 99.99% purity). The silicon target was irradiated due to the combined rotation-translation movement of the target and the nanoparticles were collected on Millipore filters (pore size 100 nm) after 36 000 laser shots. The other experimental details regarding the synthesis of QDs were presented in our previous work [26]. 

Two types of sensitive films have been made: polymeric and composite films made of polymer and QDs. For obtaining polymer films, commercial polymer solvent solutions were enhanced: 950 PMMA (MicroChem, CAS: 100-66-3) and Polyethyleneimine, low molecular weight, water-free (Aldrich, CAS: 25987-06-8). In the case of PMMA, the solvent was anisole and in the case of PDMS, the solvent was toluene. From these two solutions, compositions for composite films were also enhanced. Thus, the concentration of all the composite solutions was 0.005 g QDs in 5 mL polymer solution. These were strongly homogenized using ultrasounds. The obtained solutions were deposited by drop casting on the quartz substrate of the sensor. The volume of one drop was 20 µl. After deposition, a heat treatment at 80 °C was enhanced, with 2 h dwell. Thus, eight types of films were analyzed, as presented together with its characteristics in Table 1. 

**Figure 1:**
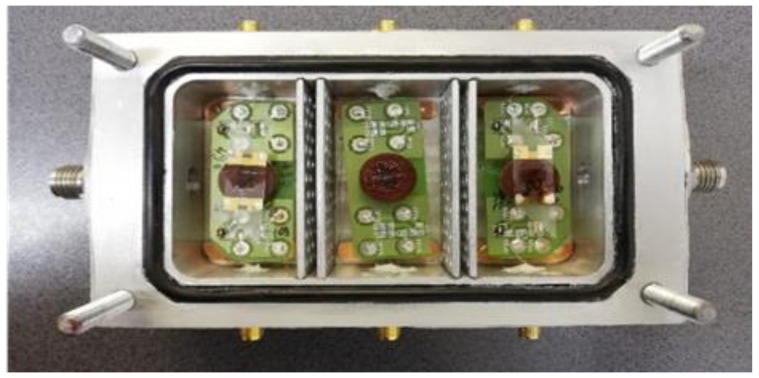
Surface Acoustic Wave (SAW)sensors in the test chamber.

The sensors were placed in the test chamber, as in Figure 1 and tested for hydrogen detection at room temperature and in synthetic air, using mass flow controllers. The hydrogen concentration was controlled by varying the flow rate of the gases from two cylinders: one with a hydrogen gas mixture (2% H_2_/98% synthetic air) and another with synthetic air (100%). The total rate was kept constant at 0.5 m–1 m for all the measurements. The sensitivity, the limit of detection (LOD), and the reversibility of the sensors were determined. Sensitivity is defined as the change in output signal obtained for a change in mass or concentration of the analyte, and represents the coefficient of the linear approximation of the frequency responses [27]. In Hz, LOD provides the signal-to-noise ratios, which in this case are defined as three times the noise level per sensitivity [2]. The noise level was determined by measuring the resonance frequency for 10 min as a maximum frequency deviation from the trend line, which was found to be around 80 Hz for all sensors.

## 3. Results and Discussion

**Figure 2:**
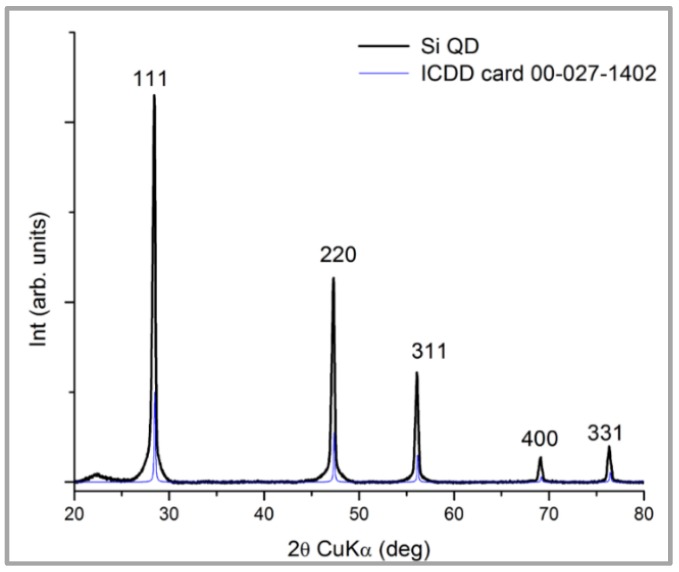
X-ray diffraction (XRD)pattern performed on the synthesized quantum dots (QDs).

From the X-ray diffraction (XRD) (Figure 2) made on the obtained powder by PLD, the presence of specific peak only for Si was noted (ICDD 00-027-1402) with a lattice constant of 0.54377 nm, almost identical to the standard, which is 0.543088.

**Figure 3:**
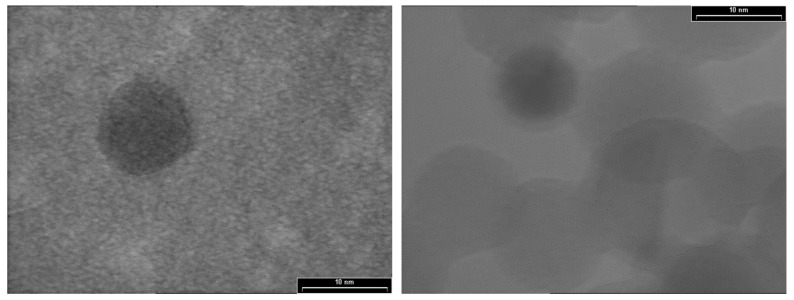
Transmission electron microscopy (TEM)images of Si/SiO_2_ QDs.

TEM images (Figure 3) indicate the formation of nanoparticles with a spherical shape and dimensions in the 2–13 nm range. Also, it can be observed that the crystalline core was covered with an amorphous layer of SiO_2_, which formed as a result of the oxidation process. The photoluminescence effect of these nanoparticles was demonstrated in our previous work [26].

The sensors developed were tested for different hydrogen concentrations, at room temperature. Table 2 shows the sensitivity and LOD. It can be seen that there are three factors that influence the sensitivity and the LOD of the sensors: the polymer used, the presence of QDs, and the heat treatment applied. PDMS is a soft polymer [28], while PMMA is a strong polymer [29]. These characteristics will influence the penetration capacity of the gas molecules in the volume of the polymeric film. After adding QDs, which leads to the growth of the specific surface of the material and to a better interaction between it and the gas molecules, the sensitivity of the PDMS QDs sensor is almost double compared to PDMS sensor. Also, it can be observed that the heat treatment applied to the films considerably improves their properties, especially for the films with QDs. This heat treatment has the role of removing organic residues present in the film from the solvents used and promoting the penetration of gas molecules in the volume of the film, due to the formation of cracks on their surface [11].

The average thickness of the deposited films was 2 µm, measured with a profilometer SURFCOM 180A (Tokyo Seimitsu, Tokyo, Japan).

**Figure 4:**
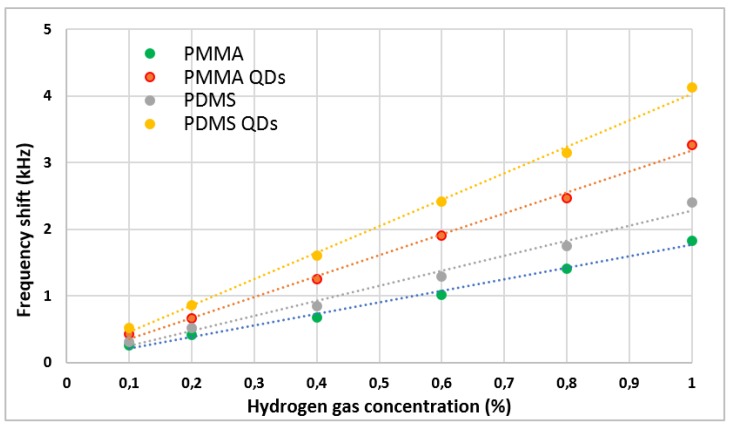
Frequency shift of the sensors for different concentrations of hydrogen.

**Figure 5:**
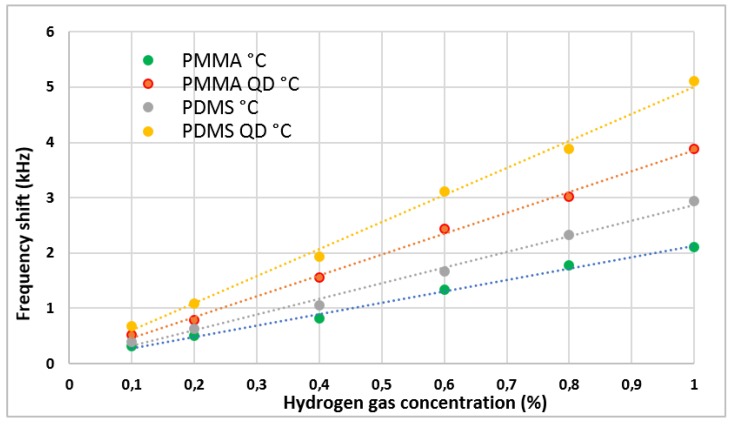
Frequency shift of the sensors after heat treatment for different concentrations of hydrogen.

The addition of QDs in the polymeric sensitive films led to an obvious increase in the sensitivity, observed both in Table 2 and Figure 4. The frequency shift for composite films was about 40% higher than those with only polymer. The heat treatment also influenced the frequency shift recorded by the sensors, as can be seen in Figure 5. After the heat treatment of the sensors with QDs, an increase of the frequency shift of about 20% was determined both for PMMA QDs and PDMS QDs. The difference of the frequency shift between the sensors with only polymer, PMMA and PDMS, and the sensors with QDs and heat treatment (PMMA QDs °C and PDMS QDs °C) was the most appreciable, with increased values of about 53% for sensors with PMMA and about 55% for those with PDMS. Of all the sensors made, the best results were obtained for those with PDMS polymer.

**Figure 6:**
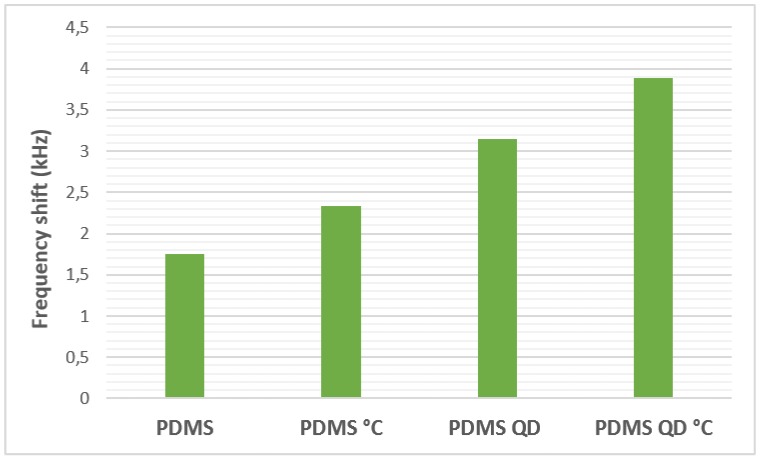
The frequency shifts for the sensor with polydimethylsiloxane (PDMS) at an 8% concentration of hydrogen.

In order to further analyze the degree of influence of the presence of QDs and of the heat treatment, Figure 6 presents the results of the frequency shifts for the sensors with PDMS at a hydrogen concentration of 8%. As can be seen, the heat treatment on the polymeric film of PDMS led to an increase in frequency shift of 0.58 kHz (about 20%). The addition of QDs in the PDMS film, however, led to an increase of 1.4 kHz, which is about two times higher than the increase observed when only the heat treatment was applied on the film (40%). When both methods of sensitivity enhancement were applied, the frequency shift increased by about 2 kHz, which means an increase of about three times greater (55%) than the increase observed when improving only with heat treatment. As a conclusion, QDs have the greatest contribution to the improvement of the sensitivity of the sensors and the heat treatment is an important parameter that helps to improve the sensors’ performance.

**Figure 7:**
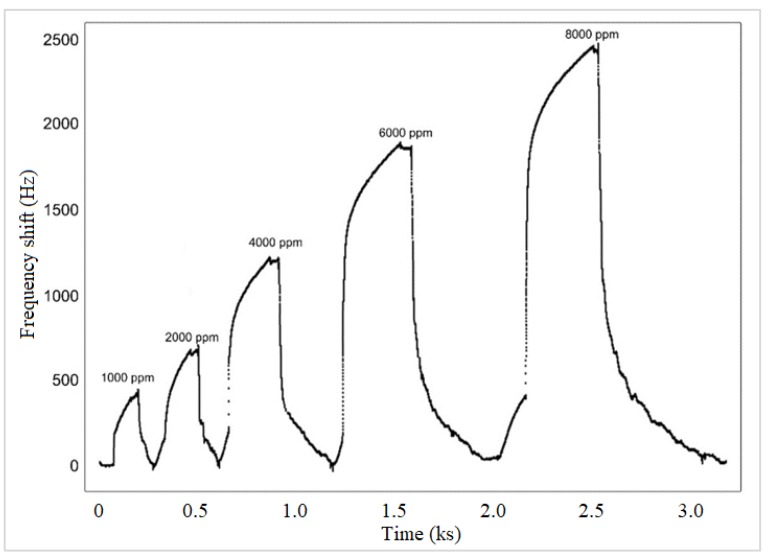
Dynamic response of sensor PMMA QDs to hydrogen for various concentrations.

Figure 7 shows the dynamic response of the PMMA QDs sensor at different analyte concentrations. As can be seen, the sensor is reversible both at lower concentrations and at higher concentrations. When polymers are used in this application, the reversibility property is very important because they can react through the functional groups with the gases to be tested. Repeating 10 measurements of the frequency deviation for each sensor, at the same concentration, yielded errors below ±3.7%.

The results obtained with PDMS QDs °C sensor are comparable with results from literature, such as those presented in Table 3. As far as can be seen, there are still challenges in obtaining sensors that operate at room temperature and which offer the highest sensitivity, with lover LOD. QDs also present a very good perspective for developing SAW sensor for other types of gases, both by varying the type of sensitive material and by using it within a composite material.

## 4. Conclusions

In this work, SAW sensors with thin polymeric and composite films were developed and studied for hydrogen detection at room temperature. The polymer films were made by PMMA and PDMS, and the composite films were made from Si QDs incorporated into the specified polymers. Also, the effect of a heat treatment at 80 °C on the frequency shift of the sensor was studied. The best results were recorded by the sensors with quantum dots, because of the large specific area that favored the mass accumulation. Also, better results were obtained after the heat treatment made on thin films. The best result was obtained by the sensor with PDMS and QDs after heat treatment. Thus, the PDMS QDs °C sensor had a LOD 452 ppm, at room temperature. These results challenge us towards a more detailed study of the use of QDs in thin films for SAW sensors and for optimizing the temperature, which will lead to much better results.

## Figures and Tables

**Table 1:** The composition of sensitive layers of the sensors studied.

Polymer	QDs	Thermal Treatment	Sensor Name
PMMA	Yes	No	PMMA QDs
		Yes	PMMA QDs °C
	No	No	PMMA
		Yes	PMMA °C
PDMS	Yes	No	PDMS QDs
		Yes	PDMS QDs °C
	No	No	PDMS
		Yes	PDMS °C

**Table 2:** Sensitivity and limit of detection (LOD) for the sensitive films. Legend: Δf, frequency change; c, hydrogen concentration.

Sensor	Sensitivity Δf/c, Hz/ppm	LOD ppm	Noise Level Hz
PMMA	0.19	1278	80
PMMA QDs	0.34	722	
PMMA °C	0.24	1037	
PMMA QDs °C	0.41	589	
PDMS	0.24	1008	
PDMS QDs	0.43	567	
PDMS °C	0.31	797	
PDMS QDs °C	0.54	452	

**Table 3:** Comparison of the results obtained in the literature for other SAW hydrogen sensors.

Sensitive Materials	Sensitivity	Limit of Detection	Working Temperature	Reference
Pd/WO_3_	0,13 Hz/ppm	4540 ppm	Room temperature	[5]
CuPc/Pd	1kHz to 5000 ppm		370 °C	[30]
ZnO nanowires	0.015 Hz/ppm	2117 ppm	Room temperature	[31]
Pd modified SnO_2_	1159 kHz to 2000 ppm	-	175 °C	[32]
ZnO nanowires	0,62	2253 ppm	Room temperature	[33]
PDMS with Si/SiO_2_ QDs	0,54	452 ppm	Room temperature	The present work
